# 1204. Evaluation Of An Antimicrobial Stewardship Scorecard For Hospitalized Patients With Community-Acquired Pneumonia

**DOI:** 10.1093/ofid/ofad500.1044

**Published:** 2023-11-27

**Authors:** Megan Klatt, Nicole Wilson, Kathryn Lamberton

**Affiliations:** The University of Kansas Health System; The University of Kansas Medical Center, Kansas City, Kansas; The University of Kansas Health System

## Abstract

**Background:**

Community-acquired pneumonia (CAP) is a common infectious disease state that provides multiple opportunities for antimicrobial stewardship interventions to optimize therapy management.

**Methods:**

This retrospective cohort study evaluated the impact of a service-level scorecard (Figure 1) with prescribing feedback on the rate of guideline concordant therapy and order set utilization in non-immunocompromised, adult patients with CAP and admitted to internal medicine and family medicine services. Scorecards were shared with teams as part of a larger antimicrobial stewardship “bundle” that included order set and guideline development, and clinician education. The primary outcome was the proportion of guideline concordant therapy defined as meeting three of our four scorecard domains: appropriate duration of therapy, appropriate empiric therapy, and inpatient IV to PO transition. Secondary outcomes included individual scorecard components of appropriate treatment duration, empiric therapy, inpatient IV to PO transition, and order set utilization.

**Figure 1:**
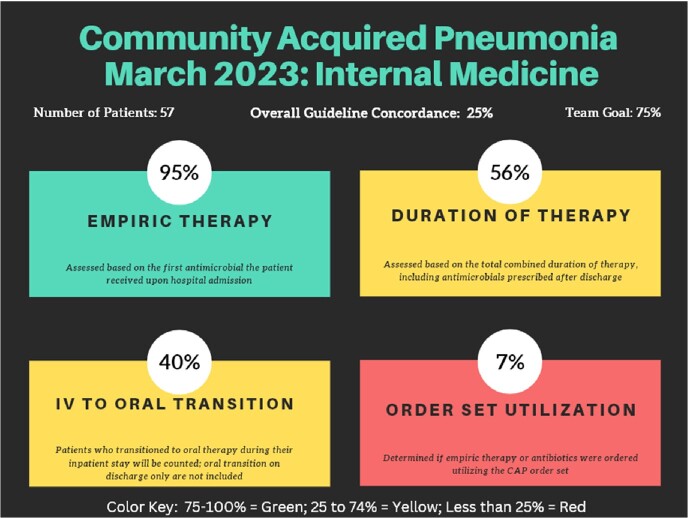
Monthly Scorecard Example

**Results:**

239 patients were included consisting of 182 in the pre-dissemination of scorecard group (December 2022 – February 2023) and 57 in the post-dissemination group (March 2023). Median age in both groups were 68-69 years with 49% males in the pre-dissemination group and 63% males in the post-dissemination group. The majority of patients were treated under the internal medicine service (Table 1). Proportion of guideline concordant therapy in the pre-dissemination group was 9% (n=16) and 25% (n=14) in the post-dissemination group (p=.007). Appropriate empiric therapy was utilized in 77% (n=140) patients in the pre-dissemination group and in 95% (n=54) in the post-dissemination group (p=.003) (Table 2).

**Figure:**
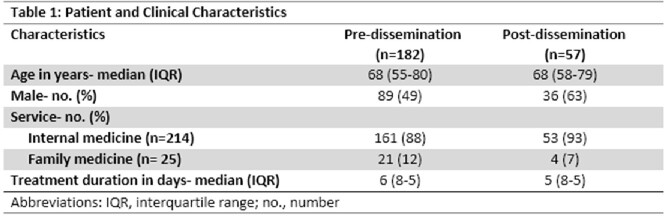

**Figure:**
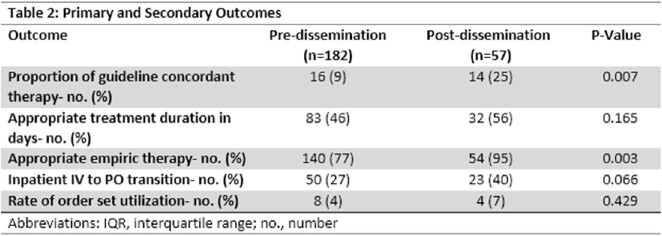

**Conclusion:**

Service-level scorecard distribution increased the rate of guideline concordant therapy amongst internal medicine and family medicine services and provided insight into creative solutions to optimize future disease state-based antimicrobial stewardship interventions.

**Disclosures:**

**All Authors**: No reported disclosures

